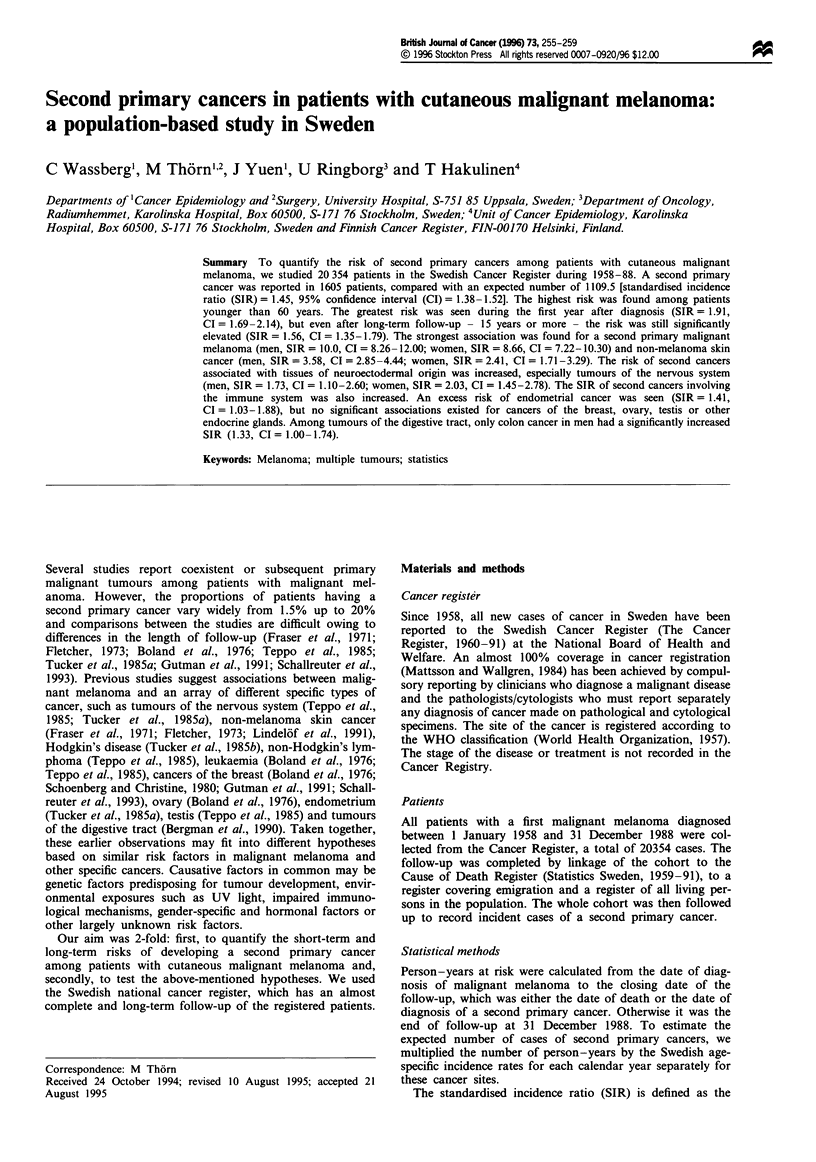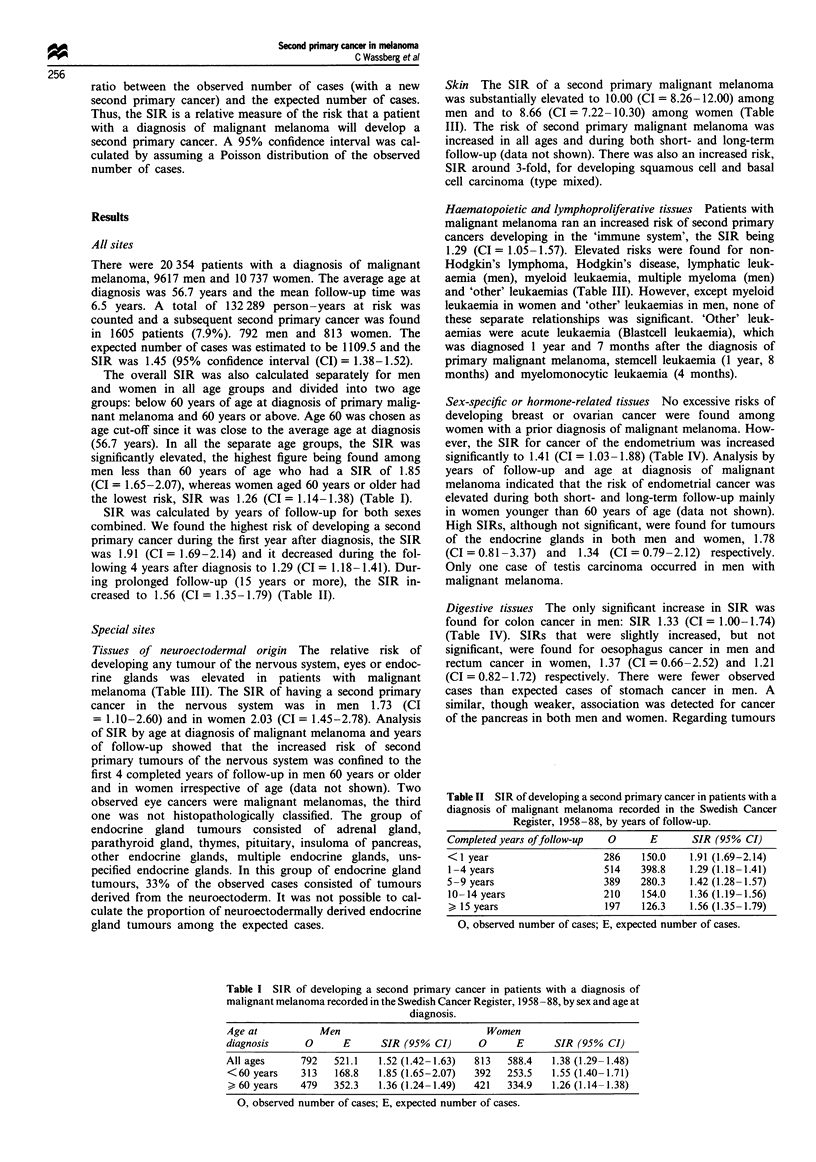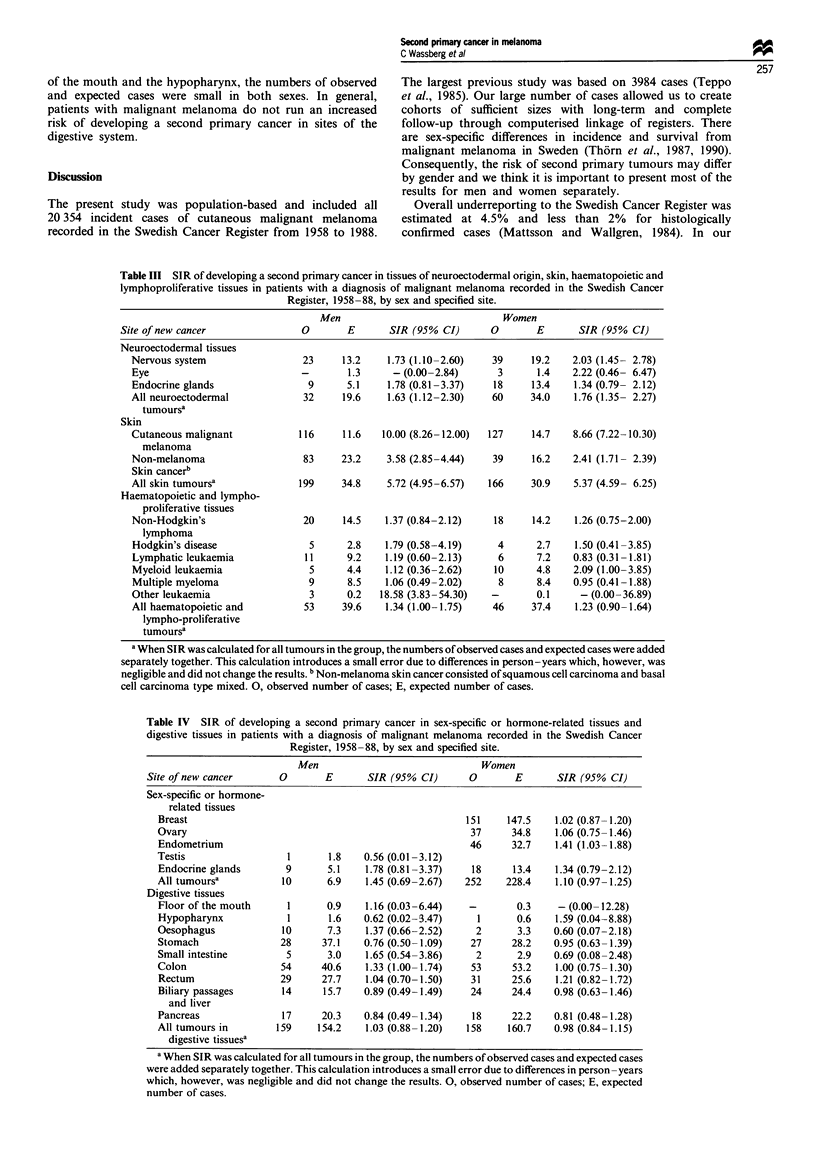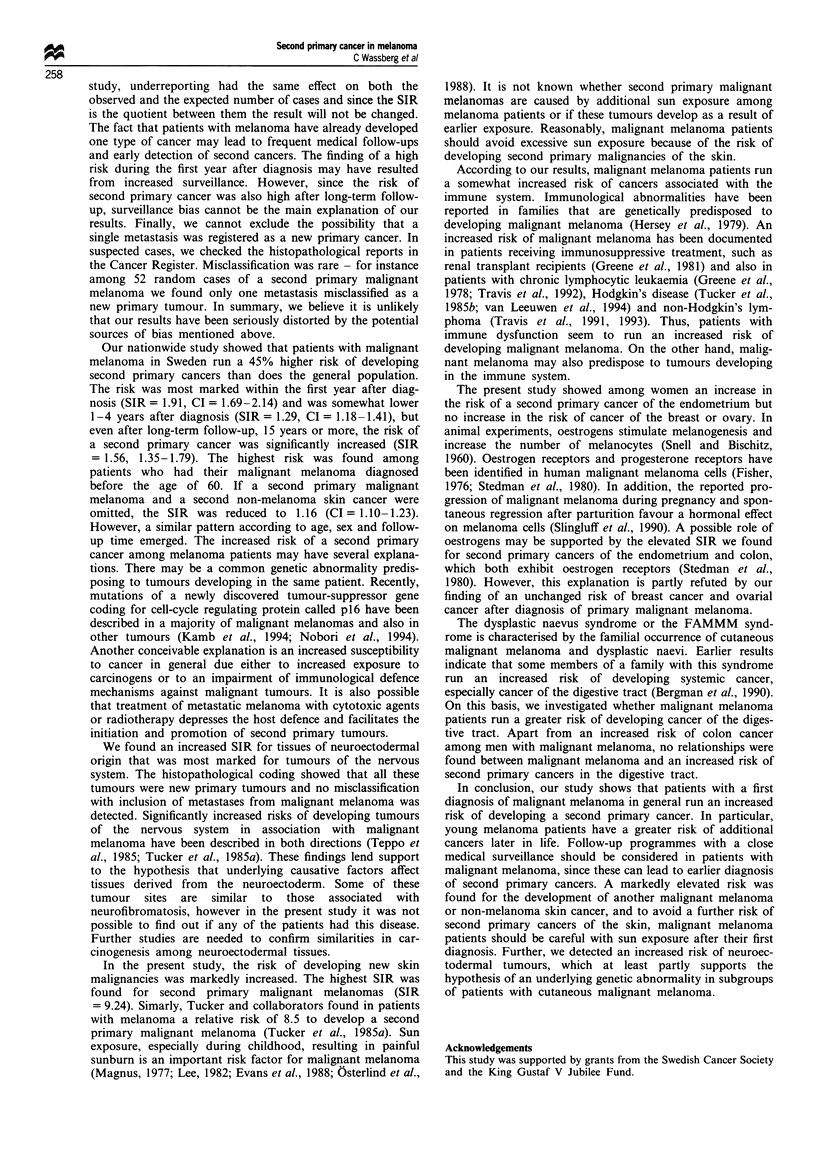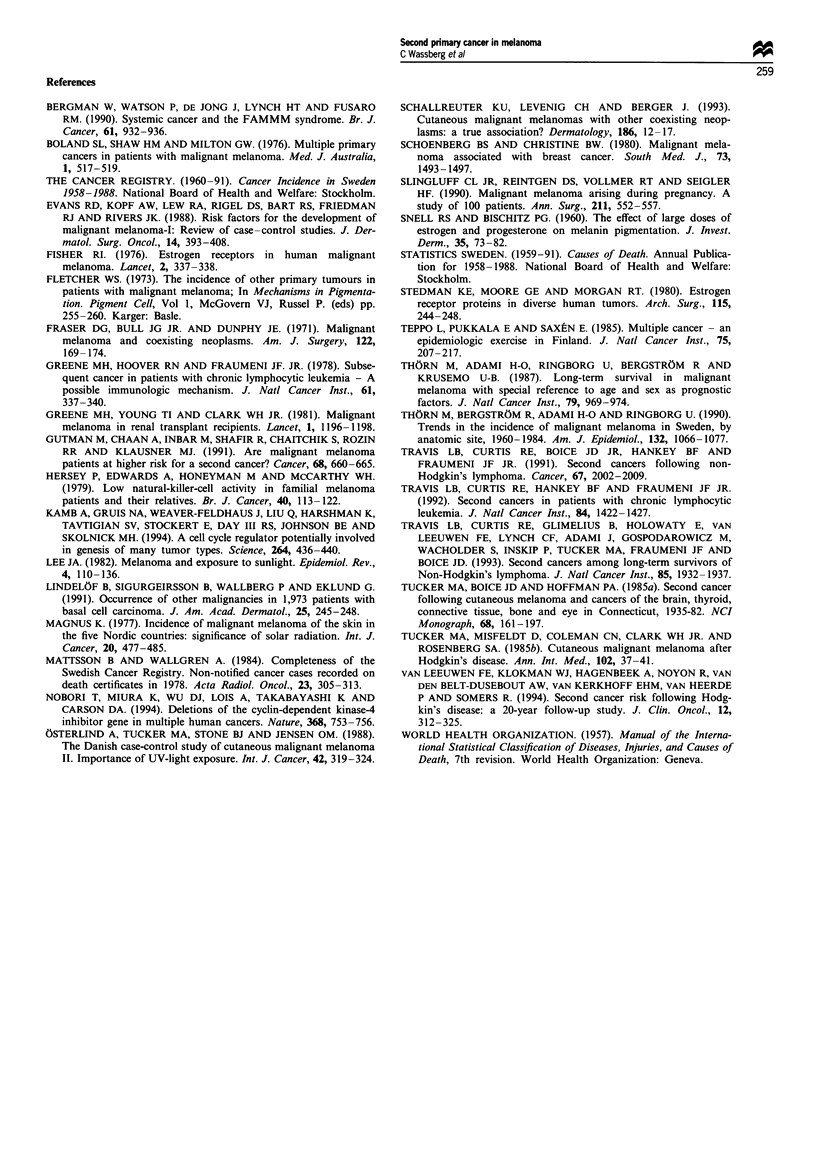# Second primary cancers in patients with cutaneous malignant melanoma: a population-based study in Sweden.

**DOI:** 10.1038/bjc.1996.45

**Published:** 1996-01

**Authors:** C. Wassberg, M. Thörn, J. Yuen, U. Ringborg, T. Hakulinen

**Affiliations:** Department of Cancer Epidemiology, University Hospital, Uppsala, Sweden.

## Abstract

To quantify the risk of second primary cancers among patients with cutaneous malignant melanoma, we studied 20,354 patients in the Swedish Cancer Register during 1958-88. A second primary cancer was reported in 1605 patients, compared with an expected number of 1109.5 [standardised incidence ratio (SIR) = 1.45, 95% confidence interval (CI) = 1.38-1.52]. The highest risk was found among patients younger than 60 years. The greatest risk was seen during the first year after diagnosis (SIR = 1.91, CI = 1.69-2.14), but even after long-term follow-up--15 years or more--the risk was still significantly elevated (SIR = 1.56, CI = 1.35-1.79). The strongest association was found for a second primary malignant melanoma (men, SIR = 10.0, CI = 8.26-12.00; women, SIR = 8.66, CI = 7.22-10.30) and non-melanoma skin cancer (men, SIR = 3.58, CI = 2.85-4.44; women, SIR = 2.41, CI = 1.71-3.29). The risk of second cancers associated with tissues of neuroectodermal origin was increased, especially tumours of the nervous system (men, SIR = 1.73, CI = 1.10-2.60; women, SIR = 2.03, CI = 1.45-2.78). The SIR of second cancers involving the immune system was also increased. An excess risk of endometrial cancer was seen (SIR = 1.41, CI = 1.03-1.88), but no significant associations existed for cancers of the breast, ovary, testis or other endocrine glands. Among tumours of the digestive tract, only colon cancer in men had a significantly increased SIR (1.33, CI = 1.00-1.74).


British Journal of Cancer (1996) 73, 255-259
© 1996 Stockton Press All rights reserved 0007-0920/96 $12.00

### Second primary cancers in patients with cutaneous malignant melanoma: a population-based study in Sweden

C Wassberg[1], M Thörn[1,2], J Yuen[1], U Ringborg[3] and T Hakulinen[4]

*Departments of [1]Cancer Epidemiology and [2]Surgery, University Hospital, S-751 85 Uppsala, Sweden; [3]Department of Oncology, Radiumhemmet, Karolinska Hospital, Box 60500, S-171 76 Stockholm, Sweden; [4]Unit of Cancer Epidemiology, Karolinska Hospital, Box 60500, S-171 76 Stockholm, Sweden and Finnish Cancer Register, FIN-00170 Helsinki, Finland.*

**Summary** To quantify the risk of second primary cancers among patients with cutaneous malignant melanoma, we studied 20 354 patients in the Swedish Cancer Register during 1958-88. A second primary cancer was reported in 1605 patients, compared with an expected number of 1109.5 [standardised incidence ratio (SIR) = 1.45, 95% confidence interval (CI) = 1.38-1.52]. The highest risk was found among patients younger than 60 years. The greatest risk was seen during the first year after diagnosis (SIR = 1.91, CI = 1.69-2.14), but even after long-term follow-up - 15 years or more - the risk was still significantly elevated (SIR = 1.56, CI = 1.35-1.79). The strongest association was found for a second primary malignant melanoma (men, SIR = 10.0, CI = 8.26-12.00; women, SIR = 8.66, CI = 7.22-10.30) and non-melanoma skin cancer (men, SIR = 3.58, CI = 2.85-4.44; women, SIR = 2.41, CI = 1.71-3.29). The risk of second cancers associated with tissues of neuroectodermal origin was increased, especially tumours of the nervous system (men, SIR = 1.73, CI = 1.10-2.60; women, SIR = 2.03, CI = 1.45-2.78). The SIR of second cancers involving the immune system was also increased. An excess risk of endometrial cancer was seen (SIR = 1.41, CI = 1.03-1.88), but no significant associations existed for cancers of the breast, ovary, testis or other endocrine glands. Among tumours of the digestive tract, only colon cancer in men had a significantly increased SIR (1.33, CI = 1.00-1.74).

**Keywords:** Melanoma; multiple tumours; statistics

Several studies report coexistent or subsequent primary malignant tumours among patients with malignant melanoma. However, the proportions of patients having a second primary cancer vary widely from 1.5% up to 20% and comparisons between the studies are difficult owing to differences in the length of follow-up (Fraser *et al.*, 1971; Fletcher, 1973; Boland *et al.*, 1976; Teppo *et al.*, 1985; Tucker *et al.*, 1985*a*; Gutman *et al.*, 1991; Schallreuter *et al.*, 1993). Previous studies suggest associations between malignant melanoma and an array of different specific types of cancer, such as tumours of the nervous system (Teppo *et al.*, 1985; Tucker *et al.*, 1985*a*), non-melanoma skin cancer (Fraser *et al.*, 1971; Fletcher, 1973; Lindelöf *et al.*, 1991), Hodgkin's disease (Tucker *et al.*, 1985*b*), non-Hodgkin's lymphoma (Teppo *et al.*, 1985), leukaemia (Boland *et al.*, 1976; Teppo *et al.*, 1985), cancers of the breast (Boland *et al.*, 1976; Schoenberg and Christine, 1980; Gutman *et al.*, 1991; Schallreuter *et al.*, 1993), ovary (Boland *et al.*, 1976), endometrium (Tucker *et al.*, 1985*a*), testis (Teppo *et al.*, 1985) and tumours of the digestive tract (Bergman *et al.*, 1990). Taken together, these earlier observations may fit into different hypotheses based on similar risk factors in malignant melanoma and other specific cancers. Causative factors in common may be genetic factors predisposing for tumour development, environmental exposures such as UV light, impaired immunological mechanisms, gender-specific and hormonal factors or other largely unknown risk factors.

Our aim was 2-fold: first, to quantify the short-term and long-term risks of developing a second primary cancer among patients with cutaneous malignant melanoma and, secondly, to test the above-mentioned hypotheses. We used the Swedish national cancer register, which has an almost complete and long-term follow-up of the registered patients.

Correspondence: M Thörn
Received 24 October 1994; revised 10 August 1995; accepted 21 August 1995

### Materials and methods

### *Cancer register*

Since 1958, all new cases of cancer in Sweden have been reported to the Swedish Cancer Register (The Cancer Register, 1960-91) at the National Board of Health and Welfare. An almost 100% coverage in cancer registration (Mattsson and Wallgren, 1984) has been achieved by compulsory reporting by clinicians who diagnose a malignant disease and the pathologists/cytologists who must report separately any diagnosis of cancer made on pathological and cytological specimens. The site of the cancer is registered according to the WHO classification (World Health Organization, 1957). The stage of the disease or treatment is not recorded in the Cancer Registry.

### *Patients*

All patients with a first malignant melanoma diagnosed between 1 January 1958 and 31 December 1988 were collected from the Cancer Register, a total of 20354 cases. The follow-up was completed by linkage of the cohort to the Cause of Death Register (Statistics Sweden, 1959-91), to a register covering emigration and a register of all living persons in the population. The whole cohort was then followed up to record incident cases of a second primary cancer.

### *Statistical methods*

Person-years at risk were calculated from the date of diagnosis of malignant melanoma to the closing date of the follow-up, which was either the date of death or the date of diagnosis of a second primary cancer. Otherwise it was the end of follow-up at 31 December 1988. To estimate the expected number of cases of second primary cancers, we multiplied the number of person-years by the Swedish age-specific incidence rates for each calendar year separately for these cancer sites.

The standardised incidence ratio (SIR) is defined as the ratio between the observed number of cases (with a new second primary cancer) and the expected number of cases. Thus, the SIR is a relative measure of the risk that a patient with a diagnosis of malignant melanoma will develop a second primary cancer. A 95% confidence interval was calculated by assuming a Poisson distribution of the observed number of cases.

### Results

### *All sites*

There were 20 354 patients with a diagnosis of malignant melanoma, 9617 men and 10 737 women. The average age at diagnosis was 56.7 years and the mean follow-up time was 6.5 years. A total of 132 289 person–years at risk was counted and a subsequent second primary cancer was found in 1605 patients (7.9%). 792 men and 813 women. The expected number of cases was estimated to be 1109.5 and the SIR was 1.45 (95% confidence interval (CI) = 1.38–1.52).

The overall SIR was also calculated separately for men and women in all age groups and divided into two age groups: below 60 years of age at diagnosis of primary malignant melanoma and 60 years or above. Age 60 was chosen as age cut-off since it was close to the average age at diagnosis (56.7 years). In all the separate age groups, the SIR was significantly elevated, the highest figure being found among men less than 60 years of age who had a SIR of 1.85 (CI = 1.65–2.07), whereas women aged 60 years or older had the lowest risk, SIR was 1.26 (CI = 1.14–1.38) (Table I).

SIR was calculated by years of follow-up for both sexes combined. We found the highest risk of developing a second primary cancer during the first year after diagnosis, the SIR was 1.91 (CI = 1.69–2.14) and it decreased during the following 4 years after diagnosis to 1.29 (CI = 1.18–1.41). During prolonged follow-up (15 years or more), the SIR increased to 1.56 (CI = 1.35–1.79) (Table II).

### *Special sites*

*Tissues of neuroectodermal origin* The relative risk of developing any tumour of the nervous system, eyes or endocrine glands was elevated in patients with malignant melanoma (Table III). The SIR of having a second primary cancer in the nervous system was in men 1.73 (CI = 1.10–2.60) and in women 2.03 (CI = 1.45–2.78). Analysis of SIR by age at diagnosis of malignant melanoma and years of follow-up showed that the increased risk of second primary tumours of the nervous system was confined to the first 4 completed years of follow-up in men 60 years or older and in women irrespective of age (data not shown). Two observed eye cancers were malignant melanomas, the third one was not histopathologically classified. The group of endocrine gland tumours consisted of adrenal gland, parathyroid gland, thymes, pituitary, insuloma of pancreas, other endocrine glands, multiple endocrine glands, unspecified endocrine glands. In this group of endocrine gland tumours, 33% of the observed cases consisted of tumours derived from the neuroectoderm. It was not possible to calculate the proportion of neuroectodermally derived endocrine gland tumours among the expected cases.

*Skin* The SIR of a second primary malignant melanoma was substantially elevated to 10.00 (CI = 8.26–12.00) among men and to 8.66 (CI = 7.22–10.30) among women (Table III). The risk of second primary malignant melanoma was increased in all ages and during both short- and long-term follow-up (data not shown). There was also an increased risk, SIR around 3-fold, for developing squamous cell and basal cell carcinoma (type mixed).

*Haematopoietic and lymphoproliferative tissues* Patients with malignant melanoma ran an increased risk of second primary cancers developing in the 'immune system', the SIR being 1.29 (CI = 1.05–1.57). Elevated risks were found for non-Hodgkin's lymphoma, Hodgkin's disease, lymphatic leukaemia (men), myeloid leukaemia, multiple myeloma (men) and 'other' leukaemias (Table III). However, except myeloid leukaemia in women and 'other' leukaemias in men, none of these separate relationships was significant. 'Other' leukaemias were acute leukaemia (Blastcell leukaemia), which was diagnosed 1 year and 7 months after the diagnosis of primary malignant melanoma, stemcell leukaemia (1 year, 8 months) and myelomonocytic leukaemia (4 months).

*Sex-specific or hormone-related tissues* No excessive risks of developing breast or ovarian cancer were found among women with a prior diagnosis of malignant melanoma. However, the SIR for cancer of the endometrium was increased significantly to 1.41 (CI = 1.03–1.88) (Table IV). Analysis by years of follow-up and age at diagnosis of malignant melanoma indicated that the risk of endometrial cancer was elevated during both short- and long-term follow-up mainly in women younger than 60 years of age (data not shown). High SIRs, although not significant, were found for tumours of the endocrine glands in both men and women, 1.78 (CI = 0.81–3.37) and 1.34 (CI = 0.79–2.12) respectively. Only one case of testis carcinoma occurred in men with malignant melanoma.

*Digestive tissues* The only significant increase in SIR was found for colon cancer in men: SIR 1.33 (CI = 1.00–1.74) (Table IV). SIRs that were slightly increased, but not significant, were found for oesophagus cancer in men and rectum cancer in women, 1.37 (CI = 0.66–2.52) and 1.21 (CI = 0.82–1.72) respectively. There were fewer observed cases than expected cases of stomach cancer in men. A similar, though weaker, association was detected for cancer of the pancreas in both men and women. Regarding tumours

**Table II** SIR of developing a second primary cancer in patients with a diagnosis of malignant melanoma recorded in the Swedish Cancer Register, 1958–88, by years of follow-up.

| *Completed years of follow-up* | *O* | *E* | *SIR (95% CI)* |
|---|---|---|---|
| < 1 year | 286 | 150.0 | 1.91 (1.69–2.14) |
| 1–4 years | 514 | 398.8 | 1.29 (1.18–1.41) |
| 5–9 years | 389 | 280.3 | 1.42 (1.28–1.57) |
| 10–14 years | 210 | 154.0 | 1.36 (1.19–1.56) |
| ⩾ 15 years | 197 | 126.3 | 1.56 (1.35–1.79) |

O, observed number of cases; E, expected number of cases.

**Table I** SIR of developing a second primary cancer in patients with a diagnosis of malignant melanoma recorded in the Swedish Cancer Register, 1958–88, by sex and age at diagnosis.

| *Age at diagnosis* | *Men* *O* | *E* | *SIR (95% CI)* | *Women* *O* | *E* | *SIR (95% CI)* |
|---|---|---|---|---|---|---|
| All ages | 792 | 521.1 | 1.52 (1.42–1.63) | 813 | 588.4 | 1.38 (1.29–1.48) |
| < 60 years | 313 | 168.8 | 1.85 (1.65–2.07) | 392 | 253.5 | 1.55 (1.40–1.71) |
| ⩾ 60 years | 479 | 352.3 | 1.36 (1.24–1.49) | 421 | 334.9 | 1.26 (1.14–1.38) |

O, observed number of cases; E, expected number of cases.

of the mouth and the hypopharynx, the numbers of observed and expected cases were small in both sexes. In general, patients with malignant melanoma do not run an increased risk of developing a second primary cancer in sites of the digestive system.

### Discussion

The present study was population-based and included all 20 354 incident cases of cutaneous malignant melanoma recorded in the Swedish Cancer Register from 1958 to 1988. The largest previous study was based on 3984 cases (Teppo *et al.*, 1985). Our large number of cases allowed us to create cohorts of sufficient sizes with long-term and complete follow-up through computerised linkage of registers. There are sex-specific differences in incidence and survival from malignant melanoma in Sweden (Thörn *et al.*, 1987, 1990). Consequently, the risk of second primary tumours may differ by gender and we think it is important to present most of the results for men and women separately.

Overall underreporting to the Swedish Cancer Register was estimated at 4.5% and less than 2% for histologically confirmed cases (Mattsson and Wallgren, 1984). In our

**Table III** SIR of developing a second primary cancer in tissues of neuroectodermal origin, skin, haematopoietic and lymphoproliferative tissues in patients with a diagnosis of malignant melanoma recorded in the Swedish Cancer Register, 1958–88, by sex and specified site.

| | *Men* | | | *Women* | | |
|---|---|---|---|---|---|---|
| *Site of new cancer* | *O* | *E* | *SIR (95% CI)* | *O* | *E* | *SIR (95% CI)* |
| Neuroectodermal tissues | | | | | | |
| Nervous system | 23 | 13.2 | 1.73 (1.10–2.60) | 39 | 19.2 | 2.03 (1.45– 2.78) |
| Eye | – | 1.3 | – (0.00–2.84) | 3 | 1.4 | 2.22 (0.46– 6.47) |
| Endocrine glands | 9 | 5.1 | 1.78 (0.81–3.37) | 18 | 13.4 | 1.34 (0.79– 2.12) |
| All neuroectodermal tumours[a] | 32 | 19.6 | 1.63 (1.12–2.30) | 60 | 34.0 | 1.76 (1.35– 2.27) |
| Skin | | | | | | |
| Cutaneous malignant melanoma | 116 | 11.6 | 10.00 (8.26–12.00) | 127 | 14.7 | 8.66 (7.22–10.30) |
| Non-melanoma Skin cancer[b] | 83 | 23.2 | 3.58 (2.85–4.44) | 39 | 16.2 | 2.41 (1.71– 2.39) |
| All skin tumours[a] | 199 | 34.8 | 5.72 (4.95–6.57) | 166 | 30.9 | 5.37 (4.59– 6.25) |
| Haematopoietic and lympho-proliferative tissues | | | | | | |
| Non-Hodgkin's lymphoma | 20 | 14.5 | 1.37 (0.84–2.12) | 18 | 14.2 | 1.26 (0.75–2.00) |
| Hodgkin's disease | 5 | 2.8 | 1.79 (0.58–4.19) | 4 | 2.7 | 1.50 (0.41–3.85) |
| Lymphatic leukaemia | 11 | 9.2 | 1.19 (0.60–2.13) | 6 | 7.2 | 0.83 (0.31–1.81) |
| Myeloid leukaemia | 5 | 4.4 | 1.12 (0.36–2.62) | 10 | 4.8 | 2.09 (1.00–3.85) |
| Multiple myeloma | 9 | 8.5 | 1.06 (0.49–2.02) | 8 | 8.4 | 0.95 (0.41–1.88) |
| Other leukaemia | 3 | 0.2 | 18.58 (3.83–54.30) | – | 0.1 | – (0.00–36.89) |
| All haematopoietic and lympho-proliferative tumours[a] | 53 | 39.6 | 1.34 (1.00–1.75) | 46 | 37.4 | 1.23 (0.90–1.64) |

[a] When SIR was calculated for all tumours in the group, the numbers of observed cases and expected cases were added separately together. This calculation introduces a small error due to differences in person–years which, however, was negligible and did not change the results. [b] Non-melanoma skin cancer consisted of squamous cell carcinoma and basal cell carcinoma type mixed. O, observed number of cases; E, expected number of cases.

**Table IV** SIR of developing a second primary cancer in sex-specific or hormone-related tissues and digestive tissues in patients with a diagnosis of malignant melanoma recorded in the Swedish Cancer Register, 1958–88, by sex and specified site.

| | *Men* | | | *Women* | | |
|---|---|---|---|---|---|---|
| *Site of new cancer* | *O* | *E* | *SIR (95% CI)* | *O* | *E* | *SIR (95% CI)* |
| Sex-specific or hormone-related tissues | | | | | | |
| Breast | | | | 151 | 147.5 | 1.02 (0.87–1.20) |
| Ovary | | | | 37 | 34.8 | 1.06 (0.75–1.46) |
| Endometrium | | | | 46 | 32.7 | 1.41 (1.03–1.88) |
| Testis | 1 | 1.8 | 0.56 (0.01–3.12) | | | |
| Endocrine glands | 9 | 5.1 | 1.78 (0.81–3.37) | 18 | 13.4 | 1.34 (0.79–2.12) |
| All tumours[a] | 10 | 6.9 | 1.45 (0.69–2.67) | 252 | 228.4 | 1.10 (0.97–1.25) |
| Digestive tissues | | | | | | |
| Floor of the mouth | 1 | 0.9 | 1.16 (0.03–6.44) | – | 0.3 | – (0.00–12.28) |
| Hypopharynx | 1 | 1.6 | 0.62 (0.02–3.47) | 1 | 0.6 | 1.59 (0.04–8.88) |
| Oesophagus | 10 | 7.3 | 1.37 (0.66–2.52) | 2 | 3.3 | 0.60 (0.07–2.18) |
| Stomach | 28 | 37.1 | 0.76 (0.50–1.09) | 27 | 28.2 | 0.95 (0.63–1.39) |
| Small intestine | 5 | 3.0 | 1.65 (0.54–3.86) | 2 | 2.9 | 0.69 (0.08–2.48) |
| Colon | 54 | 40.6 | 1.33 (1.00–1.74) | 53 | 53.2 | 1.00 (0.75–1.30) |
| Rectum | 29 | 27.7 | 1.04 (0.70–1.50) | 31 | 25.6 | 1.21 (0.82–1.72) |
| Biliary passages and liver | 14 | 15.7 | 0.89 (0.49–1.49) | 24 | 24.4 | 0.98 (0.63–1.46) |
| Pancreas | 17 | 20.3 | 0.84 (0.49–1.34) | 18 | 22.2 | 0.81 (0.48–1.28) |
| All tumours in digestive tissues[a] | 159 | 154.2 | 1.03 (0.88–1.20) | 158 | 160.7 | 0.98 (0.84–1.15) |

[a] When SIR was calculated for all tumours in the group, the numbers of observed cases and expected cases were added separately together. This calculation introduces a small error due to differences in person–years which, however, was negligible and did not change the results. O, observed number of cases; E, expected number of cases.

study, underreporting had the same effect on both the observed and the expected number of cases and since the SIR is the quotient between them the result will not be changed. The fact that patients with melanoma have already developed one type of cancer may lead to frequent medical follow-ups and early detection of second cancers. The finding of a high risk during the first year after diagnosis may have resulted from increased surveillance. However, since the risk of second primary cancer was also high after long-term follow-up, surveillance bias cannot be the main explanation of our results. Finally, we cannot exclude the possibility that a single metastasis was registered as a new primary cancer. In suspected cases, we checked the histopathological reports in the Cancer Register. Misclassification was rare – for instance among 52 random cases of a second primary malignant melanoma we found only one metastasis misclassified as a new primary tumour. In summary, we believe it is unlikely that our results have been seriously distorted by the potential sources of bias mentioned above.

Our nationwide study showed that patients with malignant melanoma in Sweden run a 45% higher risk of developing second primary cancers than does the general population. The risk was most marked within the first year after diagnosis (SIR = 1.91, CI = 1.69–2.14) and was somewhat lower 1–4 years after diagnosis (SIR = 1.29, CI = 1.18–1.41), but even after long-term follow-up, 15 years or more, the risk of a second primary cancer was significantly increased (SIR = 1.56, 1.35–1.79). The highest risk was found among patients who had their malignant melanoma diagnosed before the age of 60. If a second primary malignant melanoma and a second non-melanoma skin cancer were omitted, the SIR was reduced to 1.16 (CI = 1.10–1.23). However, a similar pattern according to age, sex and follow-up time emerged. The increased risk of a second primary cancer among melanoma patients may have several explanations. There may be a common genetic abnormality predisposing to tumours developing in the same patient. Recently, mutations of a newly discovered tumour-suppressor gene coding for cell-cycle regulating protein called p16 have been described in a majority of malignant melanomas and also in other tumours (Kamb *et al.*, 1994; Nobori *et al.*, 1994). Another conceivable explanation is an increased susceptibility to cancer in general due either to increased exposure to carcinogens or to an impairment of immunological defence mechanisms against malignant tumours. It is also possible that treatment of metastatic melanoma with cytotoxic agents or radiotherapy depresses the host defence and facilitates the initiation and promotion of second primary tumours.

We found an increased SIR for tissues of neuroectodermal origin that was most marked for tumours of the nervous system. The histopathological coding showed that all these tumours were new primary tumours and no misclassification with inclusion of metastases from malignant melanoma was detected. Significantly increased risks of developing tumours of the nervous system in association with malignant melanoma have been described in both directions (Teppo *et al.*, 1985; Tucker *et al.*, 1985*a*). These findings lend support to the hypothesis that underlying causative factors affect tissues derived from the neuroectoderm. Some of these tumour sites are similar to those associated with neurofibromatosis, however in the present study it was not possible to find out if any of the patients had this disease. Further studies are needed to confirm similarities in carcinogenesis among neuroectodermal tissues.

In the present study, the risk of developing new skin malignancies was markedly increased. The highest SIR was found for second primary malignant melanomas (SIR = 9.24). Simarly, Tucker and collaborators found in patients with melanoma a relative risk of 8.5 to develop a second primary malignant melanoma (Tucker *et al.*, 1985*a*). Sun exposure, especially during childhood, resulting in painful sunburn is an important risk factor for malignant melanoma (Magnus, 1977; Lee, 1982; Evans *et al.*, 1988; Österlind *et al.*, 1988). It is not known whether second primary malignant melanomas are caused by additional sun exposure among melanoma patients or if these tumours develop as a result of earlier exposure. Reasonably, malignant melanoma patients should avoid excessive sun exposure because of the risk of developing second primary malignancies of the skin.

According to our results, malignant melanoma patients run a somewhat increased risk of cancers associated with the immune system. Immunological abnormalities have been reported in families that are genetically predisposed to developing malignant melanoma (Hersey *et al.*, 1979). An increased risk of malignant melanoma has been documented in patients receiving immunosuppressive treatment, such as renal transplant recipients (Greene *et al.*, 1981) and also in patients with chronic lymphocytic leukaemia (Greene *et al.*, 1978; Travis *et al.*, 1992), Hodgkin's disease (Tucker *et al.*, 1985*b*; van Leeuwen *et al.*, 1994) and non-Hodgkin's lymphoma (Travis *et al.*, 1991, 1993). Thus, patients with immune dysfunction seem to run an increased risk of developing malignant melanoma. On the other hand, malignant melanoma may also predispose to tumours developing in the immune system.

The present study showed among women an increase in the risk of a second primary cancer of the endometrium but no increase in the risk of cancer of the breast or ovary. In animal experiments, oestrogens stimulate melanogenesis and increase the number of melanocytes (Snell and Bischitz, 1960). Oestrogen receptors and progesterone receptors have been identified in human malignant melanoma cells (Fisher, 1976; Stedman *et al.*, 1980). In addition, the reported progression of malignant melanoma during pregnancy and spontaneous regression after parturition favour a hormonal effect on melanoma cells (Slingluff *et al.*, 1990). A possible role of oestrogens may be supported by the elevated SIR we found for second primary cancers of the endometrium and colon, which both exhibit oestrogen receptors (Stedman *et al.*, 1980). However, this explanation is partly refuted by our finding of an unchanged risk of breast cancer and ovarial cancer after diagnosis of primary malignant melanoma.

The dysplastic naevus syndrome or the FAMMM syndrome is characterised by the familial occurrence of cutaneous malignant melanoma and dysplastic naevi. Earlier results indicate that some members of a family with this syndrome run an increased risk of developing systemic cancer, especially cancer of the digestive tract (Bergman *et al.*, 1990). On this basis, we investigated whether malignant melanoma patients run a greater risk of developing cancer of the digestive tract. Apart from an increased risk of colon cancer among men with malignant melanoma, no relationships were found between malignant melanoma and an increased risk of second primary cancers in the digestive tract.

In conclusion, our study shows that patients with a first diagnosis of malignant melanoma in general run an increased risk of developing a second primary cancer. In particular, young melanoma patients have a greater risk of additional cancers later in life. Follow-up programmes with a close medical surveillance should be considered in patients with malignant melanoma, since these can lead to earlier diagnosis of second primary cancers. A markedly elevated risk was found for the development of another malignant melanoma or non-melanoma skin cancer, and to avoid a further risk of second primary cancers of the skin, malignant melanoma patients should be careful with sun exposure after their first diagnosis. Further, we detected an increased risk of neuroectodermal tumours, which at least partly supports the hypothesis of an underlying genetic abnormality in subgroups of patients with cutaneous malignant melanoma.

**Acknowledgements**
This study was supported by grants from the Swedish Cancer Society and the King Gustaf V Jubilee Fund.

**References**

BERGMAN W, WATSON P, DE JONG J, LYNCH HT AND FUSARO RM. (1990). Systemic cancer and the FAMMM syndrome. *Br. J. Cancer*, **61**, 932–936.

BOLAND SL, SHAW HM AND MILTON GW. (1976). Multiple primary cancers in patients with malignant melanoma. *Med. J. Australia*, **1**, 517–519.

THE CANCER REGISTRY. (1960–91). *Cancer Incidence in Sweden 1958–1988*. National Board of Health and Welfare: Stockholm.

EVANS RD, KOPF AW, LEW RA, RIGEL DS, BART RS, FRIEDMAN RJ AND RIVERS JK. (1988). Risk factors for the development of malignant melanoma-I: Review of case–control studies. *J. Dermatol. Surg. Oncol.*, **14**, 393–408.

FISHER RI. (1976). Estrogen receptors in human malignant melanoma. *Lancet*, **2**, 337–338.

FLETCHER WS. (1973). The incidence of other primary tumours in patients with malignant melanoma; In *Mechanisms in Pigmentation. Pigment Cell*, Vol 1, McGovern VJ, Russel P. (eds) pp. 255–260. Karger: Basle.

FRASER DG, BULL JG JR. AND DUNPHY JE. (1971). Malignant melanoma and coexisting neoplasms. *Am. J. Surgery*, **122**, 169–174.

GREENE MH, HOOVER RN AND FRAUMENI JF. JR. (1978). Subsequent cancer in patients with chronic lymphocytic leukemia – A possible immunologic mechanism. *J. Natl Cancer Inst.*, **61**, 337–340.

GREENE MH, YOUNG TI AND CLARK WH JR. (1981). Malignant melanoma in renal transplant recipients. *Lancet*, **1**, 1196–1198.

GUTMAN M, CHAAN A, INBAR M, SHAFIR R, CHAITCHIK S, ROZIN RR AND KLAUSNER MJ. (1991). Are malignant melanoma patients at higher risk for a second cancer? *Cancer*, **68**, 660–665.

HERSEY P, EDWARDS A, HONEYMAN M AND MCCARTHY WH. (1979). Low natural-killer-cell activity in familial melanoma patients and their relatives. *Br. J. Cancer*, **40**, 113–122.

KAMB A, GRUIS NA, WEAVER-FELDHAUS J, LIU Q, HARSHMAN K, TAVTIGIAN SV, STOCKERT E, DAY III RS, JOHNSON BE AND SKOLNICK MH. (1994). A cell cycle regulator potentially involved in genesis of many tumor types. *Science*, **264**, 436–440.

LEE JA. (1982). Melanoma and exposure to sunlight. *Epidemiol. Rev.*, **4**, 110–136.

LINDELÖF B, SIGURGEIRSSON B, WALLBERG P AND EKLUND G. (1991). Occurrence of other malignancies in 1,973 patients with basal cell carcinoma. *J. Am. Acad. Dermatol.*, **25**, 245–248.

MAGNUS K. (1977). Incidence of malignant melanoma of the skin in the five Nordic countries: significance of solar radiation. *Int. J. Cancer*, **20**, 477–485.

MATTSSON B AND WALLGREN A. (1984). Completeness of the Swedish Cancer Registry. Non-notified cancer cases recorded on death certificates in 1978. *Acta Radiol. Oncol.*, **23**, 305–313.

NOBORI T, MIURA K, WU DJ, LOIS A, TAKABAYASHI K AND CARSON DA. (1994). Deletions of the cyclin-dependent kinase-4 inhibitor gene in multiple human cancers. *Nature*, **368**, 753–756.

ÖSTERLIND A, TUCKER MA, STONE BJ AND JENSEN OM. (1988). The Danish case-control study of cutaneous malignant melanoma II. Importance of UV-light exposure. *Int. J. Cancer*, **42**, 319–324.

SCHALLREUTER KU, LEVENIG CH AND BERGER J. (1993). Cutaneous malignant melanomas with other coexisting neoplasms: a true association? *Dermatology*, **186**, 12–17.

SCHOENBERG BS AND CHRISTINE BW. (1980). Malignant melanoma associated with breast cancer. *South Med. J.*, **73**, 1493–1497.

SLINGLUFF CL JR, REINTGEN DS, VOLLMER RT AND SEIGLER HF. (1990). Malignant melanoma arising during pregnancy. A study of 100 patients. *Ann. Surg.*, **211**, 552–557.

SNELL RS AND BISCHITZ PG. (1960). The effect of large doses of estrogen and progesterone on melanin pigmentation. *J. Invest. Derm.*, **35**, 73–82.

STATISTICS SWEDEN. (1959–91). *Causes of Death*. Annual Publication for 1958–1988. National Board of Health and Welfare: Stockholm.

STEDMAN KE, MOORE GE AND MORGAN RT. (1980). Estrogen receptor proteins in diverse human tumors. *Arch. Surg.*, **115**, 244–248.

TEPPO L, PUKKALA E AND SAXÉN E. (1985). Multiple cancer – an epidemiologic exercise in Finland. *J. Natl Cancer Inst.*, **75**, 207–217.

THÖRN M, ADAMI H-O, RINGBORG U, BERGSTRÖM R AND KRUSEMO U-B. (1987). Long-term survival in malignant melanoma with special reference to age and sex as prognostic factors. *J. Natl Cancer Inst.*, **79**, 969–974.

THÖRN M, BERGSTRÖM R, ADAMI H-O AND RINGBORG U. (1990). Trends in the incidence of malignant melanoma in Sweden, by anatomic site, 1960–1984. *Am. J. Epidemiol.*, **132**, 1066–1077.

TRAVIS LB, CURTIS RE, BOICE JD JR, HANKEY BF AND FRAUMENI JF JR. (1991). Second cancers following non-Hodgkin's lymphoma. *Cancer*, **67**, 2002–2009.

TRAVIS LB, CURTIS RE, HANKEY BF AND FRAUMENI JF JR. (1992). Second cancers in patients with chronic lymphocytic leukemia. *J. Natl Cancer Inst.*, **84**, 1422–1427.

TRAVIS LB, CURTIS RE, GLIMELIUS B, HOLOWATY E, VAN LEEUWEN FE, LYNCH CF, ADAMI J, GOSPODAROWICZ M, WACHOLDER S, INSKIP P, TUCKER MA, FRAUMENI JF AND BOICE JD. (1993). Second cancers among long-term survivors of Non-Hodgkin's lymphoma. *J. Natl Cancer Inst.*, **85**, 1932–1937.

TUCKER MA, BOICE JD AND HOFFMAN PA. (1985*a*). Second cancer following cutaneous melanoma and cancers of the brain, thyroid, connective tissue, bone and eye in Connecticut, 1935-82. *NCI Monograph*, **68**, 161–197.

TUCKER MA, MISFELDT D, COLEMAN CN, CLARK WH JR. AND ROSENBERG SA. (1985*b*). Cutaneous malignant melanoma after Hodgkin's disease. *Ann. Int. Med.*, **102**, 37–41.

VAN LEEUWEN FE, KLOKMAN WJ, HAGENBEEK A, NOYON R, VAN DEN BELT-DUSEBOUT AW, VAN KERKHOFF EHM, VAN HEERDE P AND SOMERS R. (1994). Second cancer risk following Hodgkin's disease: a 20-year follow-up study. *J. Clin. Oncol.*, **12**, 312–325.

WORLD HEALTH ORGANIZATION. (1957). *Manual of the International Statistical Classification of Diseases, Injuries, and Causes of Death*, 7th revision. World Health Organization: Geneva.